# Implications of Bioactive Compounds in Lifelong Disorders

**DOI:** 10.3390/life15071079

**Published:** 2025-07-07

**Authors:** Francisco Les, Guillermo Cásedas

**Affiliations:** 1 Department of Pharmacy, Faculty of Health Sciences, Universidad San Jorge, 50830 Zaragoza, Spain; fles@usj.es; 2 Instituto Agroalimentario de Aragón-IA2, CITA-Universidad de Zaragoza, 50059 Zaragoza, Spain

Over the past decade, there has been a remarkable surge in research focused on bioactive compounds, reflecting their growing importance in fields such as pharmacology, nutrition, and disease prevention [1,2,3]. According to data from PubMed, the number of publications on bioactive compounds has increased dramatically from approximately 2800 articles ten years ago to over 10,000 in recent years. This rapid growth underscores the expanding interest in understanding the biological effects, therapeutic potential, and mechanisms of action of these compounds, as well as their applications in developing novel treatments and functional foods [4,5,6,7].

Lifelong disorders are diseases or disorders that typically persist for a person’s entire life. They often have no complete cure, though many can be managed or treated to improve quality of life. These disorders usually require long-term medical care, lifestyle adjustments, or ongoing treatment. These lifelong disorders encompass genetic, neurological, metabolic or mental health disorders as well as autoimmune diseases [8,9,10,11,12,13]. Not every chronic condition is technically lifelong (some people may experience remission or late onset), but lifelong disorders are usually considered incurable, stable, or progressive throughout life.

For this reason, research on bioactive compounds emerges as an alternative motivated by their potential to offer safer, more effective, and often more natural therapeutic options [14,15]. Many lifelong disorders currently rely on long-term treatments that may cause significant side effects or have limited efficacy. Bioactive compounds—found in plants, foods, or marine organisms—often possess anti-inflammatory, antioxidant, neuroprotective, or immunomodulatory properties that could help manage disease progression, alleviate symptoms, and improve quality of life [2,16,17,18,19]. Furthermore, some bioactive compounds may target multiple pathways simultaneously, which is particularly valuable for complex, multifactorial lifelong disorders. An example of a chronic autoimmune and inflammatory disorder is Coeliac disease that develops in genetically predisposed individuals after gluten ingestion. Currently, the only effective treatment is a strict, lifelong gluten-free diet; however, challenges in maintaining dietary compliance may result in complications, emphasizing the need for additional therapeutic options. In this context, bioactive compounds, such as salvianolic acid or polyphenols from cowpea flour and sorghum bran, emerge as a promising alternative for the management of such disorders [20,21]. In this context, marine polysaccharides, due to their excellent biocompatibility, biodegradability, mucoadhesive properties, responsiveness to external stimuli, and targeting capabilities, serve as promising wall materials for oral colon-targeted delivery of polyphenols in the nutritional management of inflammatory bowel disease (IBD) [22]. Similarly, in other conditions involving the gut, such as autism spectrum disorders (ASDs), bioactive compounds like dietary polyphenols have shown potential due to their ability to modulate key pro-inflammatory and pro-oxidant signaling pathways. Since many ASD patients present gastrointestinal impairments and alterations in the gut–brain axis, polyphenols may exert therapeutic effects by influencing both gut and neurological functions. Recent preclinical and clinical studies support the potential of polyphenols in alleviating ASD symptoms, further illustrating the value of bioactive compounds as candidates for managing complex disorders with gastrointestinal and systemic involvement [23,24]. Continued research aims to better understand their mechanisms of action, optimize their bioavailability, and develop them into novel therapeutic agents or supportive treatments [25,26].

This Special Issue underscores the therapeutic potential of diverse natural products and phytochemicals across a range of pathological conditions. A systematic review of randomized controlled trials (2013–2023) suggests that cannabidiol (CBD) exhibits anxiolytic properties through mechanisms involving 5HT-1A receptor agonism and CB1 receptor modulation, although heterogeneity in study design and dosage warrants further investigation. A phytochemical review of the genus *Ruspolia* reveals antioxidant flavone glycosides and alkaloids with uncharacterized pharmacological profiles, highlighting the need for mechanistic studies. Biogenically synthesized silver nanoparticles using *Aronia melanocarpa* extracts demonstrated significant antimicrobial and antioxidant activity, mediated by phytocompounds such as polyphenols and amino acids. An investigation into *Persicaria minor* indicates that its standardized aqueous extract exerts antihypertensive effects via downregulation of the ACE/AT1R pathway, with efficacy comparable to captopril. In metabolic studies, dietary fiber formulations containing *Opuntia ficus-indica* peel improved serum glucose and triglyceride levels without adverse effects. Quercetin and luteolin were found to suppress melanoma cell proliferation and migration via GPER-mediated apoptosis and cell cycle arrest. Royal jelly conferred hepatoprotective effects against vincristine-induced toxicity by restoring antioxidant capacity and modulating JAK/STAT and PI3K/AKT/mTOR signaling pathways. The citrus flavonoid tangeretin attenuated features of diet-induced fatty liver disease, improved metabolic and hepatic markers, and restored insulin signaling, demonstrating comparable effects to metformin. A pilot randomized controlled trial evaluating an exosome-containing plant extract (ECPE) from *Ecklonia cava* and *Thuja orientalis* reported significant increases in hair density in males with androgenetic alopecia. Finally, microbial extracts derived from cacti-associated endophytes, particularly *Stenotrophomonas maltophilia*, exhibited selective cytotoxicity against glioblastoma cells, suggesting potential as a novel source of anti-glioma agents. Collectively, these studies emphasize the promising biomedical applications of natural compounds and support the need for further translational research.

More recently, bioactive compounds have continued to advance into clinical trials or serve as leads for the development of compounds that have entered clinical evaluation. This Special Issue may contribute to shed a light on new therapeutic tools in lifelong disorders.

Overall, these investigations highlight the wide-ranging therapeutic potential of bioactive compounds—such as flavonoids, polyphenols, plant extracts, microbial metabolites, and natural nanomaterials—in addressing lifelong disorders including anxiety, metabolic syndrome, neurodevelopmental and neurodegenerative diseases, cancer, and inflammatory conditions. However, to advance these promising findings into effective clinical interventions, future research should focus on several key aspects:

- Comprehensive elucidation of mechanisms of action to better understand molecular targets and pathways.
- Optimization of dosage, bioavailability, and delivery systems (e.g., nanoparticle formulations, colon-targeted carriers) to enhance therapeutic efficacy and minimize variability.
- Long-term safety and toxicity studies, particularly for chronic use.
- Well-designed, large-scale randomized controlled trials to confirm efficacy across diverse populations and disease stages.
- Standardization of bioactive compound extraction and characterization to ensure reproducibility and consistency across studies.

By addressing these gaps, bioactive compounds may increasingly serve as effective, safer, and more personalized therapeutic options for the management of lifelong and complex disorders.
